# Development of Parvifloron D-Loaded Smart Nanoparticles to Target Pancreatic Cancer

**DOI:** 10.3390/pharmaceutics10040216

**Published:** 2018-11-04

**Authors:** Ana Santos-Rebelo, Catarina Garcia, Carla Eleutério, Ana Bastos, Sílvia Castro Coelho, Manuel A. N. Coelho, Jesús Molpeceres, Ana S. Viana, Lia Ascensão, João F. Pinto, Maria M. Gaspar, Patrícia Rijo, Catarina P. Reis

**Affiliations:** 1 Centro de Investigação em Biociências e Tecnologias da Saúde (CBIOS), Universidade Lusófona de Humanidades e Tecnologias, Campo Grande 376, 1749-024 Lisboa, Portugal; ana.rebelo1490@gmail.com (A.S.-R.); catarina.g.garcia@gmail.com (C.G.); p1609@ulusofona.pt (P.R.); 2 Department of Biomedical Sciences, Faculty of Pharmacy, University of Alcalá, Ctra. A2 km33,600 Campus Universitario, 28871 Alcalá de Henares, Spain; jesus.molpeceres@uah.es; 3 Faculdade de Farmácia, Universidade de Lisboa (FFUL), Av. Prof. Gama Pinto, 1649-003 Lisboa, Portugal; carlavania@ff.ul.pt (C.E.); anacarrerabastos@gmail.com (A.B.); jfpinto@ff.ulisboa.pt (J.F.P.); mgaspar@ff.ulisboa.pt (M.M.G.); 4 iMed.ULisboa-Faculdade de Farmácia, Universidade de Lisboa, Av. Prof. Gama Pinto, 1649-003 Lisboa, Portugal; 5 Laboratory for Process Engineering, Environment (LEPABE), Department of Chemical Engineering, Faculty of Engineering, University of Porto, 4200-135 Porto, Portugal; silviac@fe.up.pt (S.C.C.); mcoelho@fe.up.pt (M.A.N.C.); 6 Centro de Química e Bioquímica (CQB), Centro de Química Estrutural (CQE), Faculdade de Ciências, Universidade de Lisboa, Campo Grande 1749-016 Lisboa, Portugal; apsemedo@fc.ul.pt; 7 Centre for Environmental and Marine Studies (CESAM), Faculdade de Ciências, Universidade de Lisboa, Campo Grande 1749-016 Lisboa, Portugal; lmpsousa@fc.ul.pt; 8 Institute of Biophysics and Biomedical Engineering (IBEB), Faculdade de Ciências, Universidade de Lisboa, 1749-016 Lisboa, Portugal

**Keywords:** *Plectranthus ecklonii*, Parvifloron D, cytotoxicity, pancreatic cancer, nanoparticles

## Abstract

Pancreatic cancer is the eighth leading cause of cancer death worldwide. For this reason, the development of more effective therapies is a major concern for the scientific community. Accordingly, plants belonging to *Plectranthus* genus and their isolated compounds, such as Parvifloron D, were found to have cytotoxic and antiproliferative activities. However, Parvifloron D is a very low water-soluble compound. Thus, nanotechnology can be a promising delivery system to enhance drug solubility and targeted delivery. The extraction of Parvifloron D from *P. ecklonii* was optimized through an acetone ultrasound-assisted method and isolated by Flash-Dry Column Chromatography. Then, its antiproliferative effect was selectivity evaluated against different cell lines (IC_50_ of 0.15 ± 0.05 μM, 11.9 ± 0.7 μM, 21.6 ± 0.5, 34.3 ± 4.1 μM, 35.1 ± 2.2 μM and 32.1 ± 4.3 μM for BxPC3, PANC-1, Ins1-E, MCF-7, HaCat and Caco-2, respectively). To obtain an optimized stable Parvifloron D pharmaceutical dosage form, albumin nanoparticles were produced through a desolvation method (yield of encapsulation of 91.2%) and characterized in terms of size (165 nm; PI 0.11), zeta potential (−7.88 mV) and morphology. In conclusion, Parvifloron D can be efficiently obtained from *P. ecklonii* and it has shown selective cytotoxicity to pancreatic cell lines. Parvifloron D nanoencapsulation can be considered as a possible efficient alternative approach in the treatment of pancreatic cancer.

## 1. Introduction

Pancreatic cancer is one of the most deadly oncologic disease and it is estimated that it will be the second most common cause of death due to cancer in the United States (USA) in 2030 [1].

This type of cancer is difficult to diagnose early, and currently, the treatment options available are very limited, being surgical resection the only potentially curative treatment. Nevertheless, surgery may be not possible in 80−90% of the cases, and long-term survival after surgical resection is very low [2,3,4].

Chemotherapy has demonstrated a positive impact on overall survival when prescribed after surgery with curative intent, and may reduce the risk of recurrence [5]. Gemcitabine and erlotinib are some examples of approved drugs in use and nab-paclitaxel has been approved in the USA and in Europe for metastasis [6]. However, chemotherapy with classical therapeutic agents has many side effects, such as nausea and vomiting, loss of appetite, hair loss, ulcers nozzles and higher chance of infection, as it promotes a shortage of white blood cells. In order to improve the long-term survival and improve the quality of life of patients with pancreatic cancer, it is imperative to find new therapeutic agents.

The use of medicinal plants and their constituents has proved their potential as clinical alternatives to synthetized drugs, leading to the discovery of new bioactive compounds [7]. These compounds have generated a strong interest in pharmacological research, towards the development of new anticancer agents. In fact, more than 50% of the compounds with different mechanisms of action used in chemotherapy are extracted from plant materials [4,6].

Many *Plectranthus* species are used as plants with medicinal interest against a variety of diseases, such as cancer. Abietane diterpenoids have been reported as the main constituents of some species in this *genus* and are responsible for its potential therapeutic value [8]. These naturally occurring compounds display a vast array of biological activities including cytotoxic and antiproliferative activities against human tumor cells [8,9]. Diterpenoids containing an abietane skeleton have proven to be strongly cytotoxic against human leukemia cells [10].

**Figure 1:**
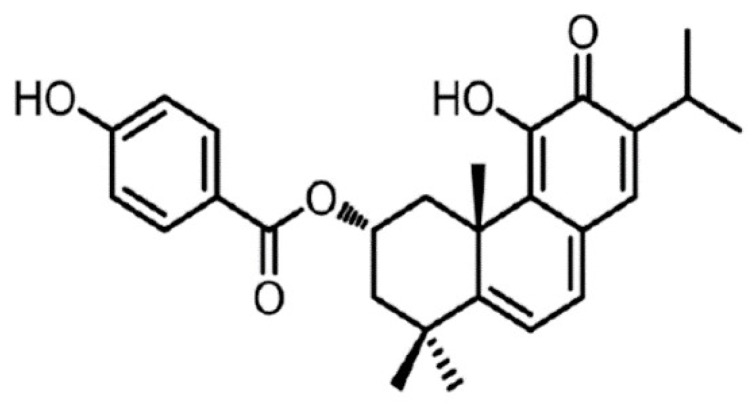
Molecular structure form Parvifloron D.

Burmistrova et al. confirmed that Parvifloron D (Figure 1) has strong cytotoxic properties against several human tumor cell lines [8]. Parvifloron D was isolated from *P. ecklonii* and thus, this plant can be associated as a good source of this abietane diterpenoid. In addition, it was also found that Parvifloron D anti-proliferative effect is generally associated with an increase in the intracellular level of Reactive Oxygen Species (ROS) that seems to play a crucial role in the apoptotic process of cells [11].

Nanotechnology has the potentiality of controlling and manipulating matter at the nanoscale by designing and engineering new systems [4]. Advances in nanoscience and nanotechnology can transform what has been done until today since new strategies will enhance and upgrade solutions to the formulation problems raised [12]. Besides improving solubility and stability of active compounds, nanoparticles may extend a formulation’s action and successfully combine active substances with different degrees of hydrophilicity [12,13,14]. Its targeting abilities to deliver drugs directly to the affected organs and tissues are another advantage of these systems that can be used in medicine [12,15].

Nanocarriers can improve the efficiency of drugs by changing their body distribution, decreasing acute toxicity, increasing their dissolution rate and in vivo stability concerning the risk of earlier metabolism and degradation [12,14,16,17].

The present study focuses on the optimization of the extraction and isolation of Parvifloron D, given its cytotoxic potential. Therefore, new approaches to target pancreatic cancer cells will be performed to improve its selectivity. Moreover, the development of a novel diterpene-encapsulated nanosystem will be done in order to optimize the Parvifloron D stability.

## 2. Materials and Methods

### 2.1. Materials

Plant material *P. ecklonii* Benth was given by the Faculty of Pharmacy of the University of Lisbon and it was collected from seeds provided by the herbarium of the National Botanical Garden of Kirstenbosch, South Africa. Voucher specimens (S/No. LISC) have been deposited in the herbarium of the Tropical Research Institute in Lisbon [8]. Acetone, hexane and ethyl acetate were supplied by VWR Chemicals (VWR international S.A.S., Briare, France); Silica was obtained from Merck (grade 60, 230–400 mesh, Merck KGaA, Darmstadt, Germany); Bovine serum albumin was purchased to Sigma-Aldrich (Steinheim, Germany). Culture media and antibiotics were obtained from Invitrogen (Life Technologies Corporation, Carlsbad, CA, USA). All cell lines were obtained from the American Type Culture Collection (LGC Standards S.L.U. Barcelona, Spain). Reagents for cell proliferation assays were purchased from Promega (Madison, WI, USA). All reagents used for the nanoparticles preparation were of analytical grade and purified water obtained by a Millipore system (Millipore, Burlington, MA, USA).

### 2.2. Extraction and Isolation

#### 2.2.1. Extraction

The whole plant-dried powdered *P. ecklonii* (197.55 g) was used to perform the Parvifloron D exhaustive extraction followed by thin-layer chromatography (TLC) (hexane: ethyl acetate, 7:3 (*v*/*v*)). The ultrasound-assisted extraction was performed using the acetone (10 × 600 mL) as the extraction solvent. The extract was obtained (28.54 g) by filtration and evaporation of acetone under vacuum (<40 °C) [9].

#### 2.2.2. Isolation

Repeated Flash-Dry Column Chromatography of *P. ecklonii* extract (25 g), over silica gel (Merck 9385, 75 g), using n-hexane: ethyl acetate mixtures of increasing polarity, allowed the isolation of pure Parvifloron D (0.882 g) [18]. The chemical structure of Parvifloron D was elucidated comparing the ^1^H-NMR spectroscopic data (Table S1: NMR data of PvD, (CDCl3, ^1^H 400 MHz, ^13^C 100 MHz; δ in ppm, J in Hz) and Table S2: Significant assignments observed on Heteronuclear Multiple Bond Correlation (HMBC) experiment for Parvifloron D) which was almost identical to those in the literature [9,19].

### 2.3. Parvifloron D Quantification by HPLC-DAD Analysis

The High-Performance Liquid Chromatography (HPLC) quantification of Parvifloron D from *P. ecklonii* extract was carried out as previously described [20]. It was used as a Liquid Chromatograph Agilent Technologies 1200 Infinity Series Liquid Chromatography (LC) System equipped with diode array detector (DAD), using a ChemStation Software (Agilent Technologies, Waldbronn, Germany) and a LiChrospher, 100 RP-18 (5 mm) column from Merck (Darmstadt, Germany). Parvifloron D was determined and quantified by injecting 20 µL of the sample at 1 mg/mL, using a gradient composed of Solution A (methanol), Solution B (acetonitrile) and Solution D (0.3% trichloroacetic acid in water) as follows: 0 min, 15% A, 5% B and 80% D; 20 min, 80% A, 10% B and 10% D; 25 min, 80% A, 10% B and 10% D. The flow rate was set at 1 mL/min. The authentic sample of Parvifloron D was run under the same conditions in methanol, and the detection was carried out between 200 and 600 nm with a diode array detector (DAD). All analyses were performed in triplicate.

### 2.4. Cell Culture and Cytotoxicity Assays

In order to evaluate Parvifloron D selectivity and antiproliferative effects against human tumor cells, different cell lines were tested: three pancreatic (BxPC3, PANC-1 and Ins1-E) and three non-pancreatic (MCF-7, HaCat and Caco-2) cell lines.

All cell lines tested, BxPC3 (human pancreas adenocarcinoma), PANC-1 (human pancreas adenocarcinoma), Ins1-E (rat pancreas insulinoma), MCF-7 (human breast cancer), HaCat (human keratinocyte) and Caco-2 (colon adenocarcinoma), are typically adherent cell cultures. Evaluations were made in different conditions regarding the different types of cells. Thus, BxPC3 cells were maintained in Roswell Park Memorial Institute (RPMI)-1640 medium with 10% heat-inactivated FBS; Ins1-E cells were maintained in RPMI medium supplemented with 10% fetal bovine serum, 100 IU/mL of penicillin, 100 μg/mL streptomycin and β-mercaptoethanol (50 µM) 1:1000; MCF-7, PANC-1 and HaCat cells were maintained in Dulbecco’s Modified Eagle’s medium (DMEM) with high-glucose (4500 mg/L), supplemented with 10% fetal bovine serum and 100 IU/mL of penicillin and 100 μg/mL streptomycin; and Caco-2 cells were maintained in RPMI medium supplemented with 10% fetal bovine serum and 100 IU/mL of penicillin and 100 μg/mL streptomycin all at 37 °C in a humidified atmosphere of 5% CO_2_ incubator.

The effects of Parvifloron D on cell growth were evaluated by different assays, namely, for BxPC3 cells, the sulforhodamine B (SRB) assay (colorimetric) was used [21] and for Ins1-E, MCF-7, PANC-1, HaCat and Caco-2 cells the MTT test was used [10]. Briefly, cells were seeded in 96-well plates (using a cell concentration of 800 cells per well for BxPC3, 1 × 10^4^ cell/mL per well for Ins1-E, MCF-7, PANC-1, HaCat and Caco-2) under normal conditions (5% CO_2_ humidified atmosphere at 37 °C) and allowed to adhere for 24 h. The cells were then incubated with Parvifloron D at different concentrations: between 0.5 and 25.0 µM for BxPC3 and to Ins1-E, MCF-7, PANC-1, HaCat and Caco-2 between 10.0 and 60.0 µM. Following this incubation period, and once the cells were analyzed through different assays, they were processed under different conditions. BxPC3 cells were fixed with 10% trichloroacetic acid for 1 h on ice, and washed and stained with 50 μL 0.4% SRB dye for 30 min. The cells were then washed repeatedly with 1% acetic acid to remove unbound dye. After, the cells were dried, and the protein-bound stain was solubilized with 10 mM Tris solution.

The SRB absorbance was measured at 560 nm using the microplate reader Model 680 (Bio-Rad, Hercules, CA, USA). The concentration that inhibits cell survival in 50% (IC_50_) was determined using the SRB assay. The absorbance of the wells containing the drug and the absorbance of the wells containing untreated cells, following a 24 h incubation period, were subsequently compared with that of the wells containing the cells that had been fixed at time zero (when Parvifloron D was added). Similarly, Ins1-E, MCF-7, PANC-1, HaCat and Caco-2 cells medium was removed, and the wells were washed with Phosphate-Buffered Saline (PBS). Then, 50 µL of a 10% MTT solution was added to the cells and the plates were incubated for 4 h. After the incubation time, 100 µL of DMSO were added to each well to solubilize the formazan crystals formed during the incubation period.

The absorbance of all samples was again measured at 570 nm using the microplate reader and IC_50_ was determined.

The cytotoxic effect was evaluated by determining the percentage of viable/death cells for each Parvifloron D studied concentration. Based on these values, the IC_50_ (Parvifloron D concentration that induces a 50% inhibition of cell growth) was calculated, according to an equation proposed by Hills and co-workers [22]. For IC_50_ determination, two concentrations, *X*_1_ and *X*_2_, and the respective cell densities, *Y*_1_ and *Y*_2_, that correspond to higher or lesser than half cell density in negative control (*Y*_0_), were established, according to the following equation:

Log IC_50_ = Log *X*_1_ + {[(*Y*_1_ − (*Y*_0_)/2)]/(*Y*_1_ − *Y*_2_)} x (Log *X*_2_ − Log *X*_1_) (1)

where, *Y*_0_/2 is the half-cell density of the negative control; *Y*_1_ is the cell density above *Y*_0_/2; *X*_1_ is the concentration corresponding to *Y*_1_; *Y*_2_ is the cell density below *Y*_0_/2; *X*_2_ is the concentration corresponding to *Y*_2_; The IC_50_ was determined by linear interpolation between *X*_1_ and *X*_2_.

### 2.5. Parvifloron D Solubility Assays

Parvifloron D solubility in PBS (pH 7.4, European Pharmacopoeia 7.0) was determined at two different temperatures, 25 °C and 37 °C, by measuring the amount of compound dissolved in a saturated solution (∼30 μg/mL) after 24 h, with constant stirring (200 rpm). Three independent measurements at each condition were conducted (*n* = 3) [23].

### 2.6. Parvifloron D Encapsulation into a Biocompatible and Hydrophilic Nanomaterial

In previous studies, Parvifloron D has showed low water-solubility, probably due to its long carbon chains and the presence of aromatic rings, giving Parvifloron D lipophilic characteristics [24,25], along with an apparent lack of selectivity to cancer cells [23]. Therefore, the encapsulation of Parvifloron D into a biocompatible and hydrophilic nanomaterial as a drug delivery system had the main objective of the achievement of optimized bioavailability and stability of the drug and thus, optimal drug loading and release properties, a long shelf life and higher therapeutic efficacy, with lower side effects [26].

Albumin was chosen as the encapsulating material to Parvifloron D due to its biocompatibility and affinity to the liver. The technology used to produce nanoparticles was the desolvation method, suitable to a wide range of polymers, especially heat-sensitives ones such as albumin, being the main advantage that it does not require an increase in temperature [27,28]. Briefly, bovine serum albumin was dissolved in purified distilled water with the pH adjusted to 8.2 with NaOH 0.1 M. Subsequently, Parvifloron D was dissolved in acetone and added to the albumin solution, which was added dropwise into a solution of absolute ethanol under magnetic stirring (500 rpm). After stirring, an opalescent suspension was spontaneously formed at room temperature. After this desolvation process, glucose in water (1.8%, *v*/*v*) was added to cross-link the desolvated albumin nanoparticles. The cross-linking process was performed under stirring of the colloidal suspension over a time period of 30 min. Measurement of pH was conducted with a pH electrode meter (827 pH lab Metrohm) calibrated daily with buffer solutions pH 4.00 ± 0.02 and 7.00 ± 0.02 (25 °C).

### 2.7. Determination of the Parvifloron D Encapsulation Efficiency by HPLC Analysis

Parvifloron D encapsulation efficiency (*EE*%) was determined using a reverse-phase HPLC chromatographic method (stationary phase—LiChrospher RP 18 (5 µm), Lichrocart 250–4.6) for the drug quantification at a detection wavelength of 254 nm. Briefly, a HPLC (Hitachi system LaCrom Elite, Column oven, Diode Array Detector (UV-Vis) (Hitachi High Technologies America, San Jose, CA, USA)) was used with a mobile phase comprising methanol and trichloroacetic acid 0.1% (80:20, *v*/*v*) (flow rate of 1.0 mL/min). Column conditions were maintained at 30 °C, with an injection volume of 20 μL and a run-time of 15 min. Measurements were carried out in duplicate and according to the described formula:

*EE* (%) = (Amount of encapsulated drug/Initial drug amount) × 100% (2)

### 2.8. In Vitro Release Studies

After determining Parvifloron D solubility, to maintain sink conditions during the in vitro release studies, empty nanoparticles and Parvifloron D-loaded nanoparticles were freeze-dried (24 h at −50 ± 2 °C, Freezone 2.5 L Benchtop Freeze Dry System, Labconco, MO, USA) and weighted according to the drug solubility. As an approximation to the blood pH, each sample of weighted nanoparticles was placed in a glass recipient, containing 250 mL of PBS (pH 7.4, European Pharmacopoeia 7.0), under constant stirring (200 rpm), in order to simulate the in vivo conditions [23]. At appropriate time intervals, aliquots of the release medium were collected from three different points of the dissolution medium, in order to obtain a homogenous collection of the sample. Nanoparticles were isolated from the supernatant by centrifugation (20,000 rpm for 15 min). The Parvifloron D amount collected from the in vitro release medium, at each time point, was determined by HPLC (see Section 2.7). The assay was conducted for 72 h, to assure that all Parvifloron D was released. (*n* = 3, mean ± SD).

### 2.9. Physical and Morphological Characterization of the Nanoparticles: Dynamic Light Scattering (DLS), Scanning Electron Microscopy (SEM) and Atomic Force Microscopy (AFM)

Freshly prepared empty nanoparticles and Parvifloron D-loaded nanoparticles were studied in terms of their structure, surface morphology, shape and size by DLS, SEM and AFM.

Physical characterization of the nanoparticles was carried out by evaluation of mean particle size, polydispersity index (PI) and zeta potential by DLS and electrophoretic mobility (Coulter nano-sizer Delsa Nano™ (Beckman Coulter, Brea, CA, USA)) of the nanoparticles’ concentrated suspension (*n* = 3).

For SEM, the aqueous suspensions containing empty and loaded nanoparticles were fixed with 2.5% glutaraldehyde in 0.1 M sodium phosphate buffer at pH 7.2 (European Pharmacopoeia 7.0) during 1 h. After centrifugation, the pellets were washed three times in the fixative buffer. Then, aliquots (10 μL) of the two samples suspensions were scattered over a round glass coverslip previously coated with poly-L-lysine and left to dry in a desiccator. Subsequently, the material was coated with a thin layer of gold and observed on a JEOL 5200LV scanning electron microscope (JEOL Ltd., Tokyo, Japan) at an accelerating voltage of 20 kV. Images were recorded digitally.

AFM images were acquired on an atomic force microscope, Multimode 8 coupled to Nanoscope V Controller, from Bruker, UK, by using peak force tapping and ScanAssist mode. In order to offer a clean and flat surface for AFM analysis, an aliquot of each sample (~30 μL) was mounted on a freshly cleaved mica sheet and left to dry before being analyzed. The images were obtained in ambient conditions, at a sweep rate close to 1 Hz, using scanasyst-air 0.4 N/m tips, from Bruker.

### 2.10. Physicochemical Characterization of Nanoparticles Interaction Analysis by Fourier Transform Infrared (FT-IR)

To study the possible interactions between Parvifloron D and bovine serum albumin polymer of the developed nanoparticles, FT-IR spectroscopy was conducted on freeze-dried nanoparticles samples and on each isolated compound, using potassium bromide (KBr). The FT-IR spectra was recorded by using a Nicolet FT-IR Spectrometer (Thermo Electron Corporation, Beverly, MA, USA) from 4000 to 400 cm^−1^, at a scanning speed of cm^−1^ for 256 scans by placing the KBr pellet on the attenuated total reflection objective. The final data is reported as a data average of 256 scans. The pellet was prepared in a ratio of 1:10 (*w*/*w*) of KBr to sample (nanoparticles or other component) and left to dry in a desiccator for 24 h before the analysis. The following samples were compared: empty nanoparticles (i.e., without Parvifloron D), Parvifloron D-loaded nanoparticles, physical mixture of Parvifloron D and bovine serum albumin (1:1, *w*/*w*), the polymer (bovine serum albumin), the cross-linking agent (glucose) and the drug (Parvifloron D).

### 2.11. Differential Scanning Calorimetry

In an attempt to check the purity of the drug and to confirm possible physicochemical interactions between nanoparticles and their raw components, thermal transformations and phase transitions of the nanoparticles were studied by calorimetry (Diferential Scanning Calorimetry, Q200, TA Instruments, New Castle, DE, USA) under a nitrogen gas flow of 50 mL/min (AirLiquide, Algés, Portugal). Samples (1–5 mg) were analyzed in hermetic aluminum pans at a heating rate of 10 °C /min from 40 to 400 °C. The endothermic and exothermic events were analyzed using TA-Universal Analysis software (Universal Analysis 2000 version 4.7A, TA Instruments, New Castle, DE, USA).

## 3. Results and Discussion

### 3.1. Extraction and Isolation

The diterpenoid Parvifloron D was isolated from a *P. ecklonii* extract in a total amount of 166.1 µg/mg, quantified by HPLC. This extraction yield of Parvifloron D with acetone was optimized when compared with Burmistrova et al. extraction (136.75 µg/mg) [8]. Here, the ultrasound-assisted extraction showed a higher extraction yield when compared with the previous described maceration method. The optimized isolation of Parvifloron D (882 mg, 0.45% on the dry plant) showed to be a successful isolation process which presented a higher yield of Parvifloron D in comparison with M Simões et al. results (0.27% on the dry plant) [9].

**Figure 2:**
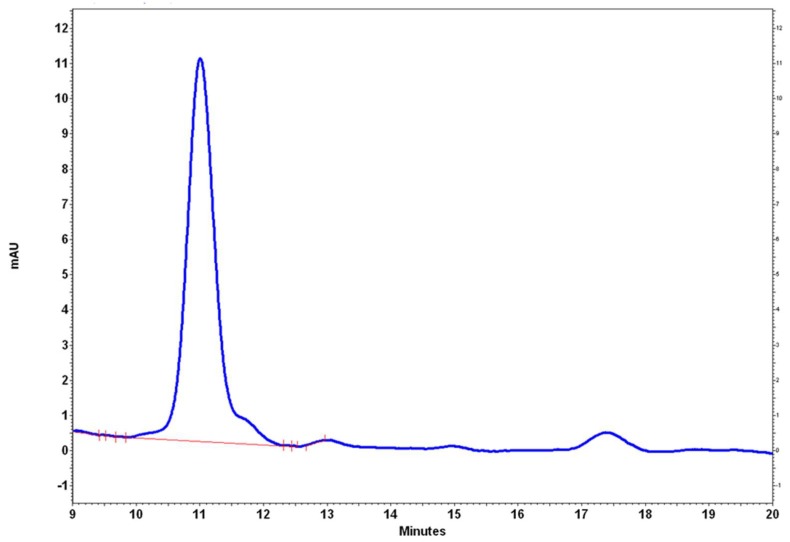
Isolated Parvifloron D spectra by High-Performance Liquid Chromatography (HPLC) analysis.

The isolated compound peak was verified by HPLC, as Figure 2 shows.

### 3.2. Parvifloron D Quantification by HPLC-DAD

**Figure 3:**
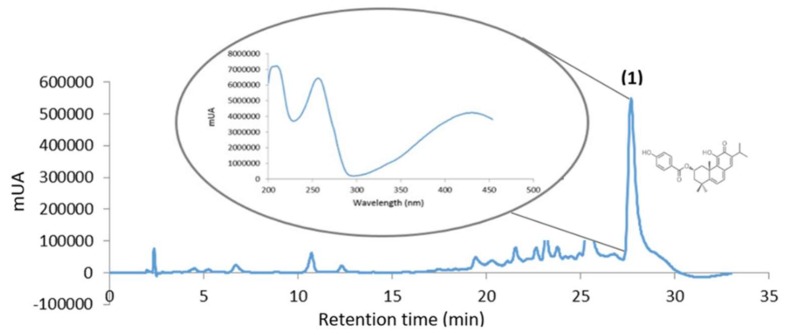
HPLC profile of *P. ecklonii* extract (254 nm): (1) Parvifloron D peak and absorption spectra.

The phytochemical analysis of *P. ecklonii* extract was performed by HPLC-DAD as represented in Figure 3. The presence of Parvifloron D was revealed (Retention Time (RT) = 27.63 min) as the principal constituent was obtained and its absorption spectra was performed, as previously described [29].

### 3.3. Cell culture and Cytotoxicity Assays

To study the cytotoxicity of free Parvifloron D, SRB and MTT assays were conducted. The results have shown that free Parvifloron D was more cytotoxic to pancreatic cell lines (BxPC3, PANC-1 and Ins1-E) than to non-pancreatic cell lines (MCF-7, Caco-2 and HaCat), displaying more selectivity to our target tumor cells than to others. Parvifloron D presented the lowest value of IC_50_ of 0.15 ± 0.05 μM for BxPC3 (human pancreatic tumor cells), and a high value of 32.1 ± 4.3 μM for Caco-2 cells (colon adenocarcinoma) according Table 1. Even in tumor cells, Parvifloron D had a higher selectivity to human tumor pancreatic cells. Cell viability in different time points, 24 h and 48 h, for Ins1-E, MCF7 and Caco-2 cells were measured and the results were added to supplementary material (Table S3—IC_50_ (µM) values in different time points, 24 h and 48 h, of different cell lines–cytotoxicity assays).

Considering some previously published results such as X. Yu et al. (IC_50_ = 0.2 μM to gemcitabine in BxPC3 cell line) [30], A. Singh et al. (IC_50_ = 123.9 μM to gemcitabine in PANC-1 cell line) [31], A. Acuna et al. (IC_50_ = 46.5 μM to PH-427 in BxPC3 cell line) [32], S. Mukai et al. (IC_50_ = 19.5 μM and 20.4 μM to gefitinib in BxPC3 and PANC-1 cell lines, respectively) [33], L. Wang et al. (IC_50_ = 39.86 μM and 83.76 μM to Pemetrexed in BxPC3 and PANC-1 cell lines, respectively) [34] or even A. Wright et al. (IC_50_ = 70.9 μM and 22.8 μM to Aphrocallistin in BxPC3 and PANC-1 cell lines, respectively) [35], we can suggest that Parvifloron D has a higher cytotoxic potential (IC_50_ = 0.15 ± 0.05) to these pancreatic tumor cells.

Despite these results and concerning the Parvifloron D mechanism of action, our group work is studding Parvifloron D-induced cell death and they have observed that Parvifloron D induces an increase in Sub-G1 and a reduction of G2/M populations in MDA-MB-231 (human breast tumor cells), as Burmistrova et al. already described in leukemia HL-60 and U-937 cells [8]. These results lead us to believe that Parvifloron D-induced cell death can be through this mechanism independently of the cell line tested. Although, this data is still in progress, and thus it has not been published yet. Nevertheless, our group has tested the internalization of polymeric nanoparticles with Parvifloron D, observing that it had occurred by endocytosis within 2 h [23]. Moreover, according to the literature, seven membrane-associated albumin-binding proteins have been discovered, namely: albondin/glycoprotein60 (gp60), glycoprotein18 (gp18), glycoprotein30 (gp30), the neonatal Fcreceptor (FcRn), heterogeneous nuclear ribonucleoproteins (hnRNPs), calreticulin, cubilin, and megalin [36]. This leads us to believe that our nanoparticles may internalize via endocytosis [37]. This assumption must be confirmed with future experiments of nanoparticles co-localization and nanoparticles cellular quantification.

### 3.4. Nanoparticles Encapsulation Efficiency by HPLC Analysis

In order to determine Parvifloron D encapsulation efficiency, a calibration curve was made using previously isolated Parvifloron D as a calibration standard. Parvifloron D standards ranging from 3 to 75 μg/mL were evaluated and a calibration curve (y = 7589.9x − 12798) was obtained with *R*^2^ = 0.999. Limit of Detection (LOD) and Limit of Quantification (LOQ) were calculated to be 2.6 μg/mL and 7.9 μg/mL, respectively.

Encapsulation efficiency (%) was determined by measuring the non-encapsulated drug. Non-encapsulated drug was measured (8.79 µg/mL) (i.e., indirect quantification) and the value obtained was subtracted from the amount of drug initially added, being the encapsulation efficiency value for Parvifloron D 91.2 ± 5.51% (mean value ± SD, *n* = 3).

### 3.5. Parvifloron D Solubility Assays and In Vitro Release Studies

HPLC studies were carried out to determine the solubility of Parvifloron D in PBS (pH 7.4) before performing in vitro release studies of Parvifloron D after entrapment into the albumin nanoparticles.

After 24h incubation in PBS pH 7.4, at two different temperatures, 25 °C and 37 °C, Parvifloron D solubility was 3.7 ± 0.8 μg/mL and 4.9 ± 0.3 μg/mL, respectively (*n* = 3).

**Figure 4:**
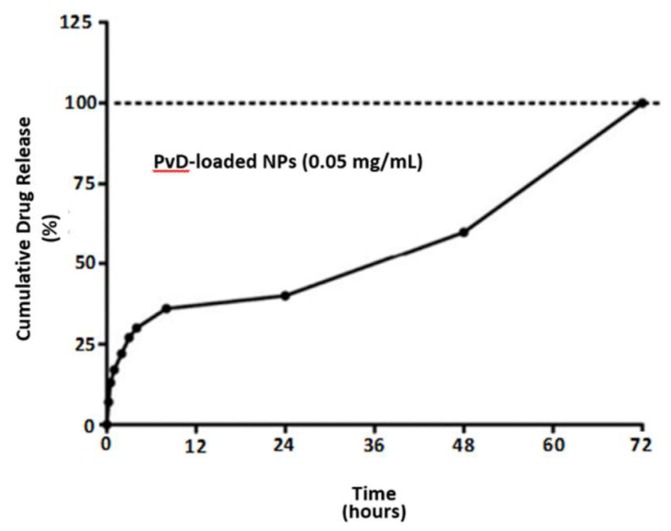
In vitro drug release of Parvifloron D-loaded nanoparticles at 0.05 mg/mL, for 72 h, in phosphate buffered saline (PBS) pH 7.4 solution. Results are expressed as mean of measurements of three independent nanoparticles lots ± Standard Deviation (SD) (*n* = 3).

Concerning the in vitro release studies, all entrapped Parvifloron D was released from albumin nanoparticles in 72 h in PBS at pH 7.4. As illustrated in Figure 4, after 24 h, approximately 40% of Parvifloron D was released from the nanoparticles and no burst release was observed. Besides, Parvifloron D degradation was evaluated as the in vitro release studies went by. The release profile was continually sustained over the assay and all of the drug was been released in less than 72 h.

### 3.6. Physical and Morphological Characterization of the Nanoparticles: DLS, AFM, SEM

In terms of mean size value, Parvifloron D-loaded nanoparticles were smaller than empty nanoparticles (165 nm (PI 0.11) and 250 nm (PI 0.37), respectively). This fact is probably due to electrostatic interactions, which may reduce and compact the particle structure. It should also be noted that there was a color change of nanoparticles from white to orange, when Parvifloron D was entrapped inside the particles. The pH value was around 8.5 in both formulations. Zeta potential was negative in both cases (−19.65 and −7.88 mV to empty nanoparticles and Parvifloron D-loaded nanoparticles, respectively) and the difference might be attributed to the presence of Parvifloron D, suggesting some interaction between the albumin and the drug, which can be related to the intrinsic charge of Parvifloron D (pKa values of 8.9, 9.9, calculated values by ChemDraw Professional).

**Figure 5:**
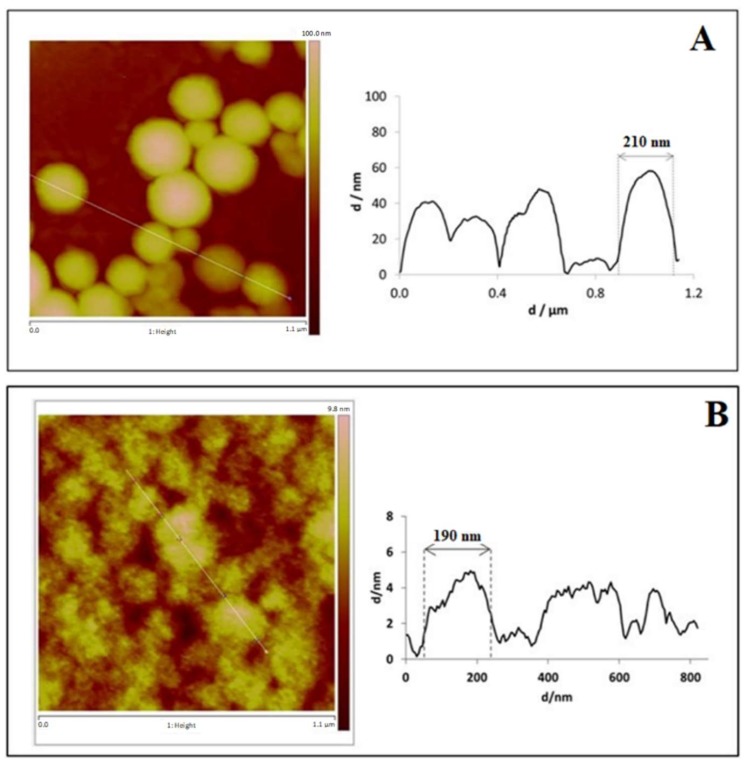
Atomic Force Microscopy (AFM) sectorial analysis of: (**A**) Albumin empty nanoparticles and (**B**) Parvifloron D-loaded nanoparticles. Particle sizes of 210 nm and 190 nm are also represented, for A and B, respectively.

**Figure 6:**
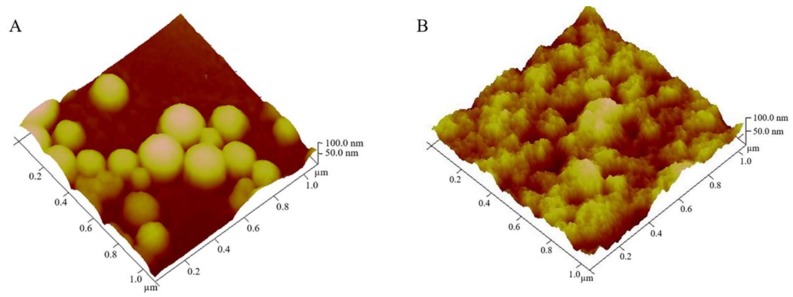
Atomic Force Microscopy (AFM) analysis 3D images of: (**A**) Albumin empty nanoparticles and (**B**) Parvifloron D-loaded nanoparticles.

AFM analysis has confirmed particles size. Figure 5 shows that the particle size of the prepared nanoparticles was approximately 210 nm and 190 nm for empty and Parvifloron D-loaded nanoparticles, respectively, as measured by DLS. AFM can offer a significant contribution to understand surface and interface properties, thus allowing for the optimization of biomaterials performance, processes, and physical and chemical properties even at the nanoscale [38]. In addition, we can notice that particles, especially the empty nanoparticles, were monodispersed. Figure 6 shows the 3D images highlighting the shape and morphology of the prepared nanoparticles.

**Figure 7:**
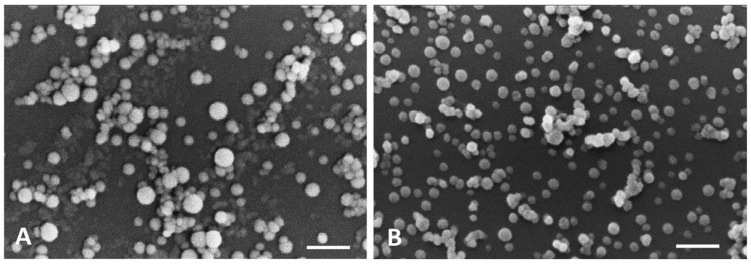
Scanning Electron Microscopy (SEM) micrographs of: (**A**) Albumin empty nanoparticles (scale bar: 1 μm) and (**B**) Parvifloron D-loaded nanoparticles (scale bar: 1 μm).

In the current study, SEM observations showed that nanoparticles in both formulations had a uniform distribution and exhibited a spherical shape with a smooth surface. It is also clearly seen that the particles size is different, the empty nanoparticles being slightly larger than the Parvifloron D-loaded nanoparticles (Figure 7). Here, SEM provides information on surface topography, size, and size distribution of nanoparticles [39].

### 3.7. Physicochemical Characterization of Nanoparticles Interaction Analysis by FT-IR

FT-IR spectra main peaks and the corresponding functional groups were identified for all tested samples. For physical mixtures and nanoparticles, the peaks were identified based on the functional groups of the raw components (wavenumbers: 4000–400 cm^−1^), as it is shown in Table 2. This analysis has demonstrated to be a useful method to interpret intra- and inter-material interactions in raw materials and their combination to obtain nanoparticles.

After the analysis of the spectra, it was confirmed the bovine serum albumin structure by the presence of specific bands for amides (I and II). Also, Parvifloron D analysis showed its specific bands, confirming its structure previously done by NMR (supplementary material). When albumin empty nanoparticles were analyzed, the same specific bands were identified in the raw bovine serum albumin spectra, although the N–H amide II band has shifted, suggesting some structure modification of bovine serum albumin chains when aggregated to provide nanoparticles. In addition, a new band at 3000 cm^−1^ appears in empty nanoparticles, probably due to the cross-linking with glucose. As for the interactions between drug and nanoparticles, it was possible to differentiate the spectra of albumin nanoparticles loaded with Parvifloron D and the physical mixture of bovine serum albumin and free Parvifloron D (at 1:1, *w*/*w*). This fact can indicate that the drug was successfully entrapped inside the nanoparticles, and observing the distinct peaks in the nanostructures analysis, some kind of drug–albumin interaction during nanoparticles formation might have occurred.

### 3.8. Differential Scanning Calorimetry

**Figure 8:**
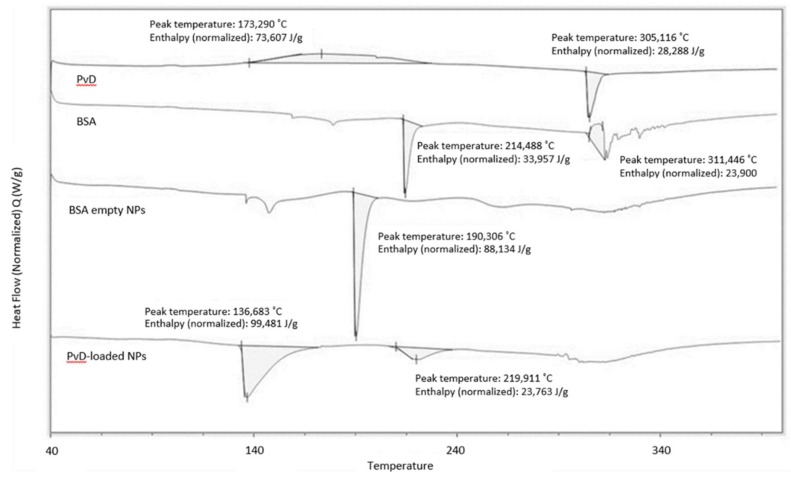
Differential Scanning Calorimetry (DSC) thermal transformations in free Parvifloron D, bovine serum albumin polymer, albumin empty nanoparticles and Parvifloron D-loaded nanoparticles, respectively. The major peak temperatures (°C) and the difference in Gibbs energy (J/g), for each sample, are also represented.

Differential scanning calorimetry was used to characterize the different properties of the developed nanosystems, such as Parvifloron D polymorphism, interactions between drug and nanoparticles and the effect on their thermal events, compared to raw materials. Figure 8 shows an exothermic peak near to 170 °C, indicating a crystallization and an endothermic peak close to 300 °C, which can represent a crystal melting, suggesting that Parvifloron D is a polymorphic drug. Anyway, to better understand the thermal behavior of Parvifloron D, more crystallographic studies have to be conducted in the future. Bovine serum albumin endothermic peaks were observed around 215 °C while exothermic peaks appeared around 310 °C. Analyzing albumin at nanoparticles form, its spectra changed, showing a lower melting point, once the endothermic peak appeared near to 190 °C, suggesting some structure modification of albumin chains to organize nanoparticles arrangement. For albumin nanoparticles loaded with Parvifloron D, the spectra show two endothermic peaks which probably represents both bovine serum albumin and Parvifloron D melting points, but due to some rearrangement between these raw materials into nanoparticles, these endothermic events had occurred at lower temperatures (136 °C and 219 °C).

## 4. Conclusions

Parvifloron D has been efficiently extracted and isolated from *P. ecklonii*. Cell cultures have shown that Parvifloron D may have more selectivity to human pancreatic tumor cells than healthy cells or breast cancer cells. Parvifloron D-loaded small and spherical nanoparticles (water soluble particles) have been formulated with high encapsulation efficiency. Those nanoparticles led to a controlled release of the drug encapsulation over 72 h. Parvifloron D nanoparticles were stable, and therefore, they can be considered a suitable and promising carrier to deliver the drug to the tumor site, improving the treatment of pancreatic cancer.

## Figures and Tables

**Table 1:** IC_50_ (± Standard deviation (SD)) values of different cell lines—cytotoxicity assays.

Cell line	IC_50_ (µM) ± SD
MCF-7 (breast cancer)	35.1 ± 2.2
HaCat (human keratinocyte)	34.3 ± 4.1
Caco-2 (Colon adenocarcinoma)	32.1 ± 4.3
INS-1E (rat pancreatic insulinoma)	21.6 ± 0.5
BxPC3 (human pancreatic adenocarcinoma)	0.15 ± 0.1
PANC-1 (human pancreatic adenocarcinoma)	11.9 ± 0.7

**Table 2:** FT-IR analysis of spectra of all tested samples (cm^−1^).

Functional Groups	O–H Carboxylic Acid (Stretching)	C–H Alkane (Stretching)	C=O Carbonyl (Stretching)	C=O Amide I (Stretching)	N–H Amide II (Bending)	C=C Aromatic (Stretching)	C–H Alkane (Bending)	C–O Alcohol (Stretching)	=C–H Alkene (Bending)
Compound									
BSA ^1^	---	---	---	1654	1590	---	---	---	---
PvD ^2^	---	2871	1693	---	---	1510	---	---	850
Glucose	3350	---	---	---	---	---	1456	1032	---
Physical mixture BSA + PvD	---	2871	1690	1658	1590	1515	---	---	---
Empty BSA-NPs ^3^	3000	---	---	1654	1540	---	---	---	---
BSA-NPs loaded With PvD	3000	2873	---	1654	1540	1540	---	---	910

^1^ BSA: Bovine serum albumin; ^2^ PvD: Parvifloron D; ^3^ NPs: nanoparticles.

## Supplementary Materials

The following are available online at http://www.mdpi.com/1999-4923/10/4/216/s1, Table S1: NMR data of PvD, (CDCl_3_, ^1^H 400 MHz, ^13^C 100 MHz; δ in ppm, J in Hz); Table S2: Significant assignments observed on HMBC experiment for PvD and Table S3: IC_50_ (µM) values in different time points, 24 h and 48 h, of different cell lines—cytotoxicity assays.
