# Supplementary material for: Development of Parvifloron D-Loaded Smart Nanoparticles to Target Pancreatic Cancer
Source: Pharmaceutics. 2018 Nov 4;10(4):216. doi: 10.3390/pharmaceutics10040216 (PMC6321128; doi:10.3390/pharmaceutics10040216)
Supplement: Supplementary file 1 [file pharmaceutics-10-00216-s001.pdf]

pharmaceutics

MDPI

Supplementary Materials: Development of Parvifloron D-loaded Smart Nanoparticles to Target Pancreatic Cancer

Ana Santos-Rebelo, Catarina Garcia, Carla Eleutério, Ana Bastos, Sílvia Castro Coelho, Manuel A. N. Coelho, Jesús Molpeceres, Ana S. Viana, Lia Ascensão, João F. Pinto, Maria M. Gaspar, Patrícia Rijo and Catarina P. Reis

Table S1. NMR data of PvD, (CDCl3,1H 400 MHz, 13C 100 MHz; δ in ppm, J in Hz).

Position	δ_H	$J_{H,H}$	δ_C	HSQC
1α	1.74 dd	(1α, 1β) 13.0	38.37	t
1β	3.76 ddd	(1α, 2β) 11.4		
2β	5.59 tt			
3α	1.56 dd			
3β	2.15 ddd		67.87	d
4	---		45.06	t
5	---		38.58	s
6	6.41 d		164.84	s
7	---		118.69	d
8	6.79 d		139.13	d
9	---		127.45	s
10	---	(1β, 2β) 4.4	127.17	s
11	---	(2β, 3α) 11.4	43.91	s
12	---	(2β, 3β) 4.4	146.40	s
13	---	(3α, 3β) 12.5	178.24	s
14	---	(6, 7) 6.9	141.61	s
15	6.96 d	(15,16(17)) 6.8	133.57	d
Me-16*	3.15 sept of d	(14,15) 0.8	26.52	d
Me-17*	1.18 d	(1β,3β) 2.4	21.84	q
Me-18	1.16 d	(2′,3′;5′,6′) 8.9	21.63	q
Me-19	1.29 s		33.03	q
Me-20	1.42 s		30.58	q
1′	1.64 s		25.52	q
2′, 6′	---		122.43	s
3′,5′	7.93 d		131.89	d
4′	6.88 d		115.23	d
7′	---		160.58	s
4′-OH	---		166.18	s
11-OH	~7.70 br			
	~7.20 br			

Table S2. Significant assignments observed on HMBC experiment for PvD.

Position	HMBC
H-2′,6′	C-7′, C-4′
H-14	C-7, C-8, C-9, C-12, C-15
H-3′,5′	C-1′, C-4′
H-7	C-5, C-6, C-8, C-9, C-14
H-6	C-4, C-5, C-7, C-8,C-10
H-2β	CO-(Ph)
H-1β	C-2, C-3, C-5, C-20
H-15	C-12, C-13, C-14, C-16(17)
H-3β	C-1, C-2, C-4, C-5, C-18, C-19
H-1α	C-2, C-3, C-5, C-9, C-20
H-3α	C-1, C-2, C-4, C-5, C-18, C-19
Me-20	C-1, C-5, C-9, C-10
Me-19	C-3, C-4, C-5, C-18
Me-18	C-3, C-4, C-5, C-19
Me-16(17)	C-13, C-15

Table S3. IC_{50} (μM) values in different time points, 24h and 48h, of different cell lines-cytotoxicity assays.

	**Ins1-E**	**MCF7**	**Caco-2**
**24 h**	21.6	35.1	32.1
**48 h**	20.9	34.7	31.9
